# Regulation and signaling pathways in cancer stem cells: implications for targeted therapy for cancer

**DOI:** 10.1186/s12943-023-01877-w

**Published:** 2023-10-18

**Authors:** Zhen Zeng, Minyang Fu, Yuan Hu, Yuquan Wei, Xiawei Wei, Min Luo

**Affiliations:** 1 Laboratory of Aging Research and Cancer Agent Target, State Key Laboratory of Biotherapy, Cancer Center, National Clinical Research Center for Geriatrics, West China Hospital, Sichuan University, No. 17, Block 3, Southern Renmin Road, Chengdu, Sichuan 610041 P.R. China; 2 Department of Pediatric Nephrology Nursing, Key Laboratory of Birth Defects and Related Diseases of Women and Children, Ministry of Education, West China Second Hospital, Sichuan University, No. 17, Block 3, Southern Renmin Road, Chengdu, Sichuan 610041 P.R. China

**Keywords:** CSCs, Regulatory networks, Cancer therapy

## Abstract

Cancer stem cells (CSCs), initially identified in leukemia in 1994, constitute a distinct subset of tumor cells characterized by surface markers such as CD133, CD44, and ALDH. Their behavior is regulated through a complex interplay of networks, including transcriptional, post-transcriptional, epigenetic, tumor microenvironment (TME), and epithelial-mesenchymal transition (EMT) factors. Numerous signaling pathways were found to be involved in the regulatory network of CSCs. The maintenance of CSC characteristics plays a pivotal role in driving CSC-associated tumor metastasis and conferring resistance to therapy. Consequently, CSCs have emerged as promising targets in cancer treatment. To date, researchers have developed several anticancer agents tailored to specifically target CSCs, with some of these treatment strategies currently undergoing preclinical or clinical trials. In this review, we outline the origin and biological characteristics of CSCs, explore the regulatory networks governing CSCs, discuss the signaling pathways implicated in these networks, and investigate the influential factors contributing to therapy resistance in CSCs. Finally, we offer insights into preclinical and clinical agents designed to eliminate CSCs.

## Introduction

Tumorigenesis and tumor progression are considered as complex and progressive processes that involve multiple levels of response and the accumulation of mutations. In the past several decades, many studies have emerged in the field of oncology regarding this topic. These studies have focused on tumor cells, the tumor microenvironment, tumor heterogeneity, etc., among which tumor heterogeneity is closely associated with tumorigenesis and the malignancy of tumors. As one of the theories to explain the underlying mechanism of tumor heterogeneity, cancer stem cells (CSCs) have received much attention [1].

CSCs were first found in the mid-1990s as a group of malignant tumor cells with the potential of self-renewal and differentiation, which were closely related to the tumorigenesis, metastasis and therapy resistance of tumors. They were identified in the blood of leukemia patients as a small specific subpopulation of cells which could initiate leukemia in immune-deficient mice [2]. In numerous studies, CSCs have consistently demonstrated their remarkable capacities for self-renewal, differentiation, sphere formation, and proliferation across various cancer types. These abilities are of significant relevance in the context of tumorigenesis. Moreover, CSCs possess the potential to drive tumor metastasis and confer resistance to therapy, thus playing pivotal roles in advancing tumor progression [3, 4]. Therefore, the study of CSCs is critical for understanding tumorigenesis and tumor progression, and many breakthroughs have already been made. To date, cell surface markers have been used to distinguish initial mutant cell populations of multiple tumors, including brain, prostate, breast, melanoma, lung and liver cancers [5–10]. However, it is still a challenge to find prospective markers to label CSCs in CSC-related studies. Generally, CSCs express CD133, CD44, EpCAM and ALDH [11, 12]. Nevertheless, CSC markers are slightly different across distinct cancer types due to tumor heterogeneity.

To gain a comprehensive understanding of CSCs and the intricate regulatory networks governing them, this review provides a comprehensive summary of the transcriptional, posttranscriptional, epigenetic modifications, tumor microenvironment (TME), and epithelial-mesenchymal transition (EMT) regulation of CSCs. Additionally, we delve into the signaling pathways intricately involved in these regulatory networks and explore influential factors contributing to therapy resistance in CSCs. Given that CSCs are increasingly recognized as promising targets in cancer treatment, we also spotlight CSC-associated targeting agents.

## The origin of CSCs

Cancer stem cells (CSCs) are a subgroup of tumor cells that were first identified in leukemia in 1994. Dick and Bonnet isolated CSCs from leukemia and successfully differentiated this cell population into various hierarchies of leukemia cells in immune-deficient mice and showed that CSCs lead to the development of leukemia [13]. Increasing evidence has proved CSCs in a variety of solid tumors and these CSCs have subsequently demonstrated the potential of tumor-propagating and cell differentiation [14]. Breast cancer is the first solid tumor type in which CSCs were shown to exist [15]. Many other solid tumors have also been proven to contain CSCs, including colon cancer [16], pancreatic cancer [17] and brain cancers [18]. Besides, the presence of CSCs in various solid tumors exhibits considerable variability. Accordingly, the origin of CSCs is one of the hot topics in CSC research and is still elusive.

Various theories were proposed for the origin of stem cells, among which adult stem cells (ASCs)-origin theory and tumor cell-origin theory are the most mentioned [19]. For ASCs-origin theory in intestinal tumors, Dagmar Beier et al. had discovered that ASCs lost the APC gene in the long-term accumulation of transforming events, leading to carcinogenesis and the potential development of CSCs [20]. Tumor cell-origin theory indicated that CSCs originated from the stem-like tumor cells organized by the tumor heterogeneity [21]. Tumor heterogeneity refers to the fact that the cells of the tumor population itself exhibit phenotypic and functional differences [22]. In most cases, the different phenotypes of CSCs were a result of the tumor heterogeneity [23]. Conversely, CSCs were also identified as one of the primary factors contributing to tumor heterogeneity [24]. Furthermore, researches have shown that the potential mechanism by which tumor cells transform into CSCs involves genetic reprogramming or dynamic state switching [19]. However, there are several other possible origins of CSCs, including embryonal rest, somatic mutation, the cell fusion hypothesis, metabolic reprogramming, etc. [25].

## Biological characteristics of CSCs

With gradual understanding of CSC characteristics, some breakthroughs have been made in tumor research. However, some CSC-related clinical problems in cancer treatments need to be further solved. Therefore, understanding the biological characteristics of CSCs is of great significance for exploring tumorigenesis and tumor development.

Self-renewal and differentiation are two representative characteristics of CSCs that could lead to tumorigenesis. Similar to ASCs, CSCs undergo both symmetric and asymmetric divisions stochastically with regulatory signaling pathways [26]. In symmetric division, one CSC divides into two to undergo self-renewal to replenish the CSC pool. Zhang et al. found that hTERT^high^ cells in prostate cancer exhibit stemness characteristics of CSCs by significantly increasing the proportion of symmetrically divided cells and realizing constant cell self-renewal [27]. Alternatively, asymmetric division produces transit-amplifying cells that terminally differentiate into tumorigenic potential cells and multilineage cells after stimulation. In glioblastoma, the endothelial differentiation function of CSCs contributes to tumor vasculature and promotes angiogenesis [28]. Additionally, CSCs also have sphere-formation and proliferation abilities. Studies had demonstrated that CSCs were distributed stochastically within a tumor and formed spheres even in serum deprivation [3, 29].

Surface markers play a crucial role in providing essential information for the understanding and investigation of CSCs. Over years of research, common surface markers have been well identified in CSCs across various tumor types. Generally, CSCs exhibit the expression of CD133, CD44, EpCAM, and ALDH in most cancers [11, 12]. Beyond these widely recognized markers, CSCs also display other non-classical markers such as CK17 and CD49f. For instance, CK17, a cytokeratin, has been reported to serve as a marker for CSCs in cervical cancer when co-expressed with OCT-4, NANOG, and SOX2 [30]. Additionally, CD49f, also known as Integrin Alpha 6, has emerged as a novel biomarker for CSCs. It is a transmembrane glycoprotein found in various tumors, including brain tumors, hepatocellular carcinoma (HCC), and lung cancer [31, 32]. However, it’s important to acknowledge that due to tumor heterogeneity, the surface markers of CSCs can exhibit slight variations in different types of cancer. For instance, Olfm4 and Ascl2 are highly expressed in gastrointestinal cancer but are absent in some hematological carcinomas [33]. CSCs from hematological carcinoma are usually positive for CD34, CD123, and CD33 but negative for CD38, CD90, CD117 and HLA-DR [34]. Brain cancers mostly express A2B5 and L1CAM, whereas cytokeratin 19 and OV-6 are specific markers for CSCs in HCC [35–37].

## Regulatory networks of CSCs

**Fig. 1:**
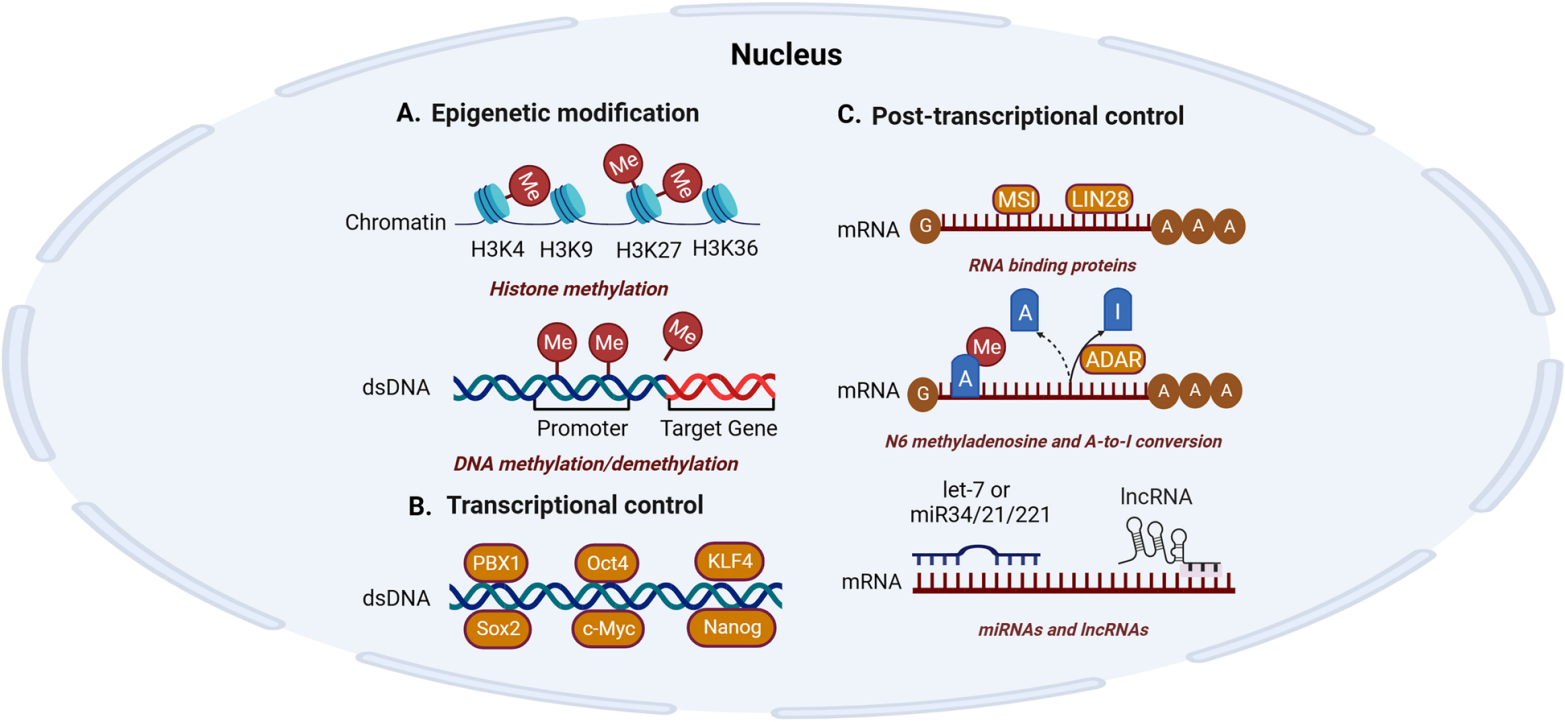
Epigenetic modification, transcriptional control and posttranscriptional control of CSCs. Epigenetic modification, transcriptional control, and posttranscriptional control are three critical mechanisms within the CSC regulatory networks. These regulatory mechanisms play pivotal roles in maintaining CSC stemness, CSC-associated tumor metastasis, and CSC-associated therapy resistance. **A** The figure illustrates how methyl groups modify DNA and histones, influencing downstream gene expression at the epigenetic level. **B** Transcriptional control in CSCs is primarily attributed to six specific transcription factors: PBX1, Oct4, Sox2, c-Myc, KLF4, and Nanog. **C** Posttranscriptional control factors encompass RNA-binding proteins, N6-methyladenosine, A-to-I conversion, miRNAs, and lncRNAs

**Fig. 2:**
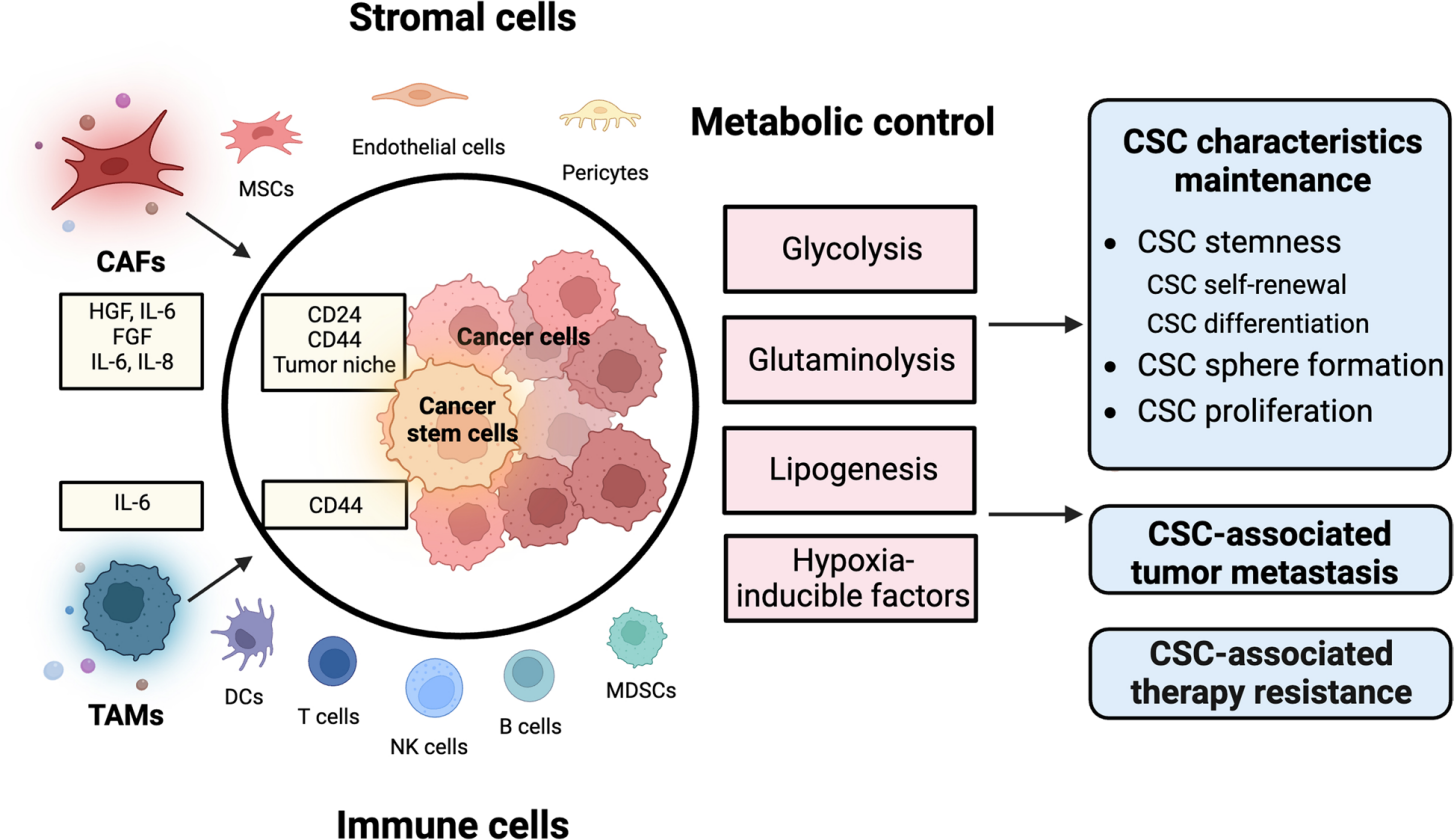
Tumor microenvironment control of CSCs. Various nontumor cells, including stromal cells and immune cells, and metabolic control which exist in the tumor microenvironment participate in CSC characteristic maintenance, CSC-associated tumor metastasis and CSC-associated therapy resistance. Cancer-associated fibroblasts (CAFs) are the most representative stromal cells, and tumor-associated macrophages (TAMs) are the most essential immune cells. They contribute to CSC characteristics maintenance by secreting particular cytokines. The detailed cytokines and corresponding factors that these cytokines influenced were all shown in the yellow boxes. In term of metabolic control which could participate in CSC characteristic maintenance, glycolysis, glutaminolysis, lipogenesis and hypoxia-inducible factors are the most representative four parts (red boxes)

Due to the inherent nature of stem cells, CSCs are proficient in self-renewal and differentiation. Additionally, they share some similar regulatory networks, such as transcription factors and posttranscriptional and epigenetic control. In addition, CSC characteristics are inseparable from complex interactions with the microenvironment. This section introduces the regulatory networks of CSCs based on five perspectives: transcriptional control, posttranscriptional control, epigenetic modification control, TME control and EMT control (Figs. 1 and 2).

### Transcriptional control of CSCs

Transcription factors (TFs) are defined as a group of protein molecules whose unique binding to genes activate a vital process, transcription, and thereby, they inhibit or enhance gene expression [38]. To date, aberrant expression of TFs has been identified in cancer cell uncontrolled proliferation, metastasis, angiogenesis and survival [39–43]. Notably, numerous TFs drive CSC-specific characteristics, such as self-renewal and differentiation [44]. Among these key stemness TFs, six specific TFs participate specifically in the transcriptional control of CSCs: Oct4, Sox2, c-Myc, KLF4, Nanog and PBX1 [45–47]. In the following section, we will introduce each of them (Fig. 1).

#### Oct4

Oct4 (also called POU5F1 or Oct3) is a putative TF involved in CSC characteristic regulation. Both upregulation and downregulation of Oct4 are implicated in CSC self-renewal, CSC-associated tumor metastasis and CSC-associated therapy resistance.

Overexpressed Oct4 has been found in CSCs in clinical tumor samples, such as oral cavity squamous cell carcinomas, pancreatic cancer and glioma, which supports CSC self-renewal [48–50]. Moreover, the high Oct4 expression observed in CSCs from lung cancer not only maintains self-renewal but also promotes CSC-associated tumor metastasis [51]. Oct4-overexpressing CSCs transactivate the M-CSF promoter to upregulate M-CSF secretion, thereby resulting in tumor metastasis [52]. In addition, EMT-associated signals are positively correlated with high Oct4 expression, which also promotes CSC-associated tumor metastasis in lung cancer, as well as in HCC [53, 54]. Furthermore, Stella Chai et al. demonstrated that in HCC, Oct4 expression directly influences CSC-associated therapy resistance [55]. This phenomenon also exists in melanoma and cervical cancer [56, 57]. Conversely, Oct4 downregulation leads to gradual loss of stemness characteristics [58]. After the knockdown of the Oct4 gene, the likelihood of malignant transformation of CSCs from pancreatic cancer was significantly reduced [59].

#### Sox2

SRY (sex determining region Y)-Box-2 (Sox2) is a transcription factor expressed by CSCs [60]. Evidence is mounting that Sox2 expression is required for the sphere-formation ability of CSCs, CSC proliferation and CSC-related chemotherapeutic resistance [61, 62].

CSCs have strong sphere-formation ability, which has been proven decades ago. High Sox2 expression is observed in nearly half of the basal cell-like breast carcinomas and is associated with sphere-formation ability and CSC proliferation [63]. Furthermore, Sox2 expression is crucial for the proliferation of CSCs in lung cancer and glioma, as it sustains the bidirectional transition between the stem-like state and the differentiated state [64, 65]. Other studies have shown that Sox2 deletion results in the blockade of tumorigenesis and deletion of CSC proliferation genes [66]. Barone C et al. demonstrated that oligodendroglioma initiation and CSC proliferation were strongly arrested by knocking down Sox2 [67]. Moreover, Sox2 knockdown-dependent cell cycle arrest and a decrease in tumoroids have been shown in glioblastoma and breast cancer [68, 69]. In addition to CSC proliferation, Sox2 regulates CSC-associated therapy resistance. Increasing evidence has illustrated that in breast cancer, CSC resistance to tamoxifen, an ER antagonist, is attributed to the activation of Sox2 [70].

#### c-Myc

In normal cell activities, the Myc family plays a fundamental role in cell metabolism, the cell cycle, and cell differentiation. The Myc family includes c-Myc, L-Myc and N-Myc, which have different functions [71]. Since c-Myc is most closely related to CSCs, this section only focuses on how c-Myc regulates CSCs in cancers. The dysregulation of c-Myc could influence CSC stemness maintenance, CSC-associated tumor metastasis and therapy resistance [72].

Superoxide dismutase (SOD2) is a downstream target gene of c-MYC, which regulates cell stemness characteristics. In tongue squamous cell carcinoma, c-MYC combines with SOD2 and drives CSC generation [73–75]. In addition, increased c-Myc expression maintains CSC stemness and induces CSC-associated tumor metastasis through EMT in breast cancer [74]. Data have demonstrated that the overexpression of c-Myc can induce the ‘awakening’ of dormant CSCs and directly regulate downstream genes to activate EMT, leading to CSC-associated tumor metastasis in nasopharyngeal cancer [75]. While promoting the stemness of CSCs, c-Myc also increases the therapy resistance of CSCs. According to Jun‐Nian Zhou et al., blocking c-Myc results in CSCs from HCC becoming more sensitive to chemical agents [76].

#### KLF4

KLF4 is a TF that regulates diverse cellular processes, such as the cell cycle and differentiation. However, KLF4 is a bifunctional TF in human cancers. In the past few years, KLF4 has been described as an anticancer factor. Studies have demonstrated that KLF4 performs a tumor suppression function in gastrointestinal cancers [77, 78], T-cell acute lymphoblastic leukemia (T-ALL) [79], lung cancer [80], meningioma [81] and bladder cancer [82].

KLF4 was found to also act as an oncogene to promote carcinogenesis by affecting CSC stemness maintenance and CSC-associated tumor metastasis. In osteosarcoma and glioma, CSCs acquire a higher self-renewal and sphere-formation ability through the KLF4-activated MAPK signaling pathway [83, 84]. The same process has been observed in pancreatic cancer. Kress TR et al. showed that KLF4 overexpression also promoted CSC-associated tumor metastasis in pancreatic cancer [85]. The mechanism was demonstrated for the first time in 2017: KLF4 could induce perivascular cell plasticity, which promotes premetastatic niche formation for CSCs [86]. Moreover, CSCs can stabilize KLF4 expression by promoting the deubiquitinating process of KLF4 and further enhance CSC-associated tumor metastasis [87]. Conversely, the suppression of KLF4 directly decreased CSC-associated tumor metastasis from the breast to the brain [88].

#### Nanog

Nanog is also a CSC-associated TF that was first discovered in embryonic stem cells. Notably, Nanog is expressed at a low level in differentiated cells but is overexpressed in stem cells [89]. It plays a central role in CSC characteristic maintenance, CSC-associated metastasis and therapy resistance in cancers.

Data have shown that the suppression of Nanog is detrimental to CSC self-renewal, sphere-formation ability, and CSC generation in glioma [90]. Alternatively, the role of Nanog overexpression in maintaining CSC characteristics has been reflected in renal, ovarian and liver cancers [91–93]. In breast cancer, high-level expression of Nanog can not only maintain CSC stemness but also promote CSC-associated tumor metastasis [94]. Liu L et al. showed that in non-small cell lung cancer (NSCLC), Nanog regulated downstream signaling pathways and protein expression to promote the EMT process, which promoted CSC-associated tumor metastasis [53]. Furthermore, overexpression of Nanog also results in CSC-associated cancer therapy resistance. Emerging evidence has demonstrated that Nanog^high^ CSCs are insensitive to gemcitabine, salinomycin and cisplatin treatments [53, 95].

#### PBX1

PBX1, a transcription factor, has been identified as a key player in both tumorigenesis and the self-renewal of CSCs [96]. Jung et al. discovered that PBX1 played a role in maintaining the characteristics of CSCs in ovarian cancer [97]. Additionally, PBX1 has been reported as a regulator of CSC self-renewal and contributes to CSC characteristics maintenance in leukemia [98, 99]. These findings suggest that PBX1 could potentially serve as a novel target for CSC therapy.

### Posttranscriptional control of CSCs

In addition to TFs, posttranscriptional control can also maintain the characteristics of CSCs and regulate CSCs. Posttranscriptional control refers to the regulation of gene expression at the RNA level, these RNAs are mainly referred to as mRNAs currently [100, 101]. Abnormalities in posttranscriptional control can lead to uncontrolled cell proliferation, vascular sprouting, EMT and other tumorigenic processes [102]. Recently, posttranscriptional control of CSCs in cancers has attracted extensive attention. Among the diverse methods of posttranscriptional control, RNA-binding proteins (RBPs), adenosine modification and noncoding RNAs have been proved to be important (Fig. 1).

#### RNA-binding proteins (RBPs)-mediated control of mRNA

RNA-binding proteins (RBPs) are of the utmost importance in tumorigenic processes. They bind to mRNA molecules once transcription initiates and regulate subsequent processing. RBPs not only act on mRNA cleavage, splicing, capping and modification but also regulate cellular stability and protein translation [103], which can also be used by tumors. To date, thousands of RBPs have been discovered in several tumors and play a role in tumor development [104, 105]. This section will introduce two key RBPs in the control of mRNA in CSCs: MSI and LIN28.

##### MSI

MSI, which regulates sensory organ precursor cells to divide asymmetrically, was identified in Drosophila. The MSI family includes MSI1 and MSI2, both of which impact CSC characteristic maintenance [106]. Data have also shown the multiple roles of MISI1 in CSC self-renewal, proliferation and CSC-associated therapy resistance [107].

Argonaute2 (AGO2) is a MIS1-binding partner that binds to MSI1 in response to environmental stress and influences cell fate. Chen HY et al. suggested that in glioblastoma and pancreatic ductal adenocarcinoma, the MSI1/AGO2 complex repressed downstream mRNA by binding to its 3’UTR or coding sequence, which allowed CSCs to maintain self-renewal and proliferation [108]. MSI1 overexpression also promotes the proliferation of CSCs in colorectal cancer (CRC). In addition, the latest research on CRC highlighted the role of 5-fluorouracil (5-FU) in inducing CSC-associated therapy resistance by upregulating MSI1 [109, 110]. In glioblastoma, overexpression of MSI1 modifies transcripts of checkpoint proteins to hyperactivate the DNA damage repair mechanism, which results in CSC-associated irradiation resistance [111].

Similar to MSI1, MSI2 also contributes to CSC self-renewal and CSC-associated therapy resistance. Emerging evidence has shown that MSI2 plays an important role as a posttranscriptional regulator in hematologic cancer [112]. In leukemia, the increased RNA binding activity of MSI2 is positively correlated with CSC self-renewal ability [113, 114]. Fang T et al. demonstrated that MSI2 upregulated CSC-related TFs in HCC, which maintained the stemness of CSCs [115]. In addition, the MSI2 protein has been proven to impede CSC sensitivity to chemotherapy and radiotherapy in ovarian cancer [116].

##### LIN28

Initially identified in Caenorhabditis elegans as a developmental regulator, LIN28 plays a role in various normal physiological processes, including cell development and proliferation. It exists in two paralog forms: LIN28a and LIN28b [117]. Emerging evidence suggests that LIN28 can regulate mRNA translation by binding to a ‘GGAGA’ motif, thereby influencing RNA splicing and processing. This is correlated with the maintenance of CSC characteristics and CSC-associated tumor metastasis [118].

Scientists have shown that LIN28 facilitates the expression of stemness-related TFs at the posttranscriptional level in several cancers, which could induce and maintain the stemness of CSCs [119, 120]. Yes-associated protein 1 (YAP1), which is the main downstream effector of the Hippo signaling pathway, is a newly discovered target of LIN28. Hailin Zou et al. have shown that LIN28 upregulates the expression of YAP1 to maintain CSC stemness and promote tumor growth in triple-negative breast cancer (TNBC) by inducing the mRNA decay of YAP1 upstream kinases [121, 122]. Furthermore, LIN28 alters the expression levels of vimentin and cadherins in breast cancer, which promote the sphere-formation ability of CSCs and CSC-associated tumor metastasis, respectively [123].

#### Adenosine modification-mediated control of mRNA

Apart from RBPs, adenosine modification of mRNA is another type of posttranscriptional control that maintains the stemness of CSCs and regulates carcinogenesis. Adenosine modification of mRNA refers to mRNA editing at the adenosine site, which changes the sequence information. N6-methyladenosine (m^6^A), adenosine-to-inosine (A-to-I) conversion and 5-methylcytosine (m^5^C) are three main forms of adenosine modification. However, the relationship between m^5^C and CSCs is uncertain. Therefore, this review focuses on m^6^A and A-to-I conversion in the remaining content.

##### N6-methyladenosine (m^6^A)

N6-methyladenosine (m^6^A) is methylation that occurs in the N6-position of adenosine, which is the most prevalent internal modification of mRNA. The action of m^6^A depends on three types of regulators, including m^6^A methyltransferase (METTLE), m^6^A demethylases, and m^6^A recognizer (the YTH and IMP families) [124]. Since m^6^A modifications are necessary for regulating cellular processing, it is not surprising that they are linked to CSC stemness maintenance, as well as CSC-associated therapy resistance.

Both m^6^A methylation and m^6^A demethylation can modify mRNAs at the posttranscriptional level to maintain CSC stemness. In terms of m^6^A methylation, reports have illustrated that YTHDF2 recognizes m^6^A installed by METTLE on the mRNA of several TFs to maintain CSC stemness [125–128]. In addition, suppressor of cytokine signaling 2 (SOCS2) is another target of METTLE for CSC stemness maintenance [129]. As a cytokine-inducible negative regulator, SOCS2 promotes CSC self-renewal and differentiation in HCC in a YTHDF2-dependent manner [130]. As for m^6^A demethylation, emerging evidence suggests that ALKBH5 removes m^6^A from FOXM1 mRNA in glioblastoma and maintains CSC stemness [117]. Furthermore, m^6^A demethylation shifts the alternative splicing of BCLX and NCOR2, which also contributes to glioma CSC self-renewal and tumor outgrowth [131–133]. Moreover, ALKBH5-dependent m^6^A demethylation of the TACC3 transcript is frequently modified in acute myeloid leukemia (AML), resulting in CSC stemness maintenance [134, 135].

Apart from functions in CSC stemness maintenance, m^6^A modification can also regulate CSC-associated therapy resistance. Scientists have suggested that m^6^A clearance decay induces CSC-associated radio-resistance in glioma [136, 137]. Furthermore, m^6^A clearance in leukemia induced by m^6^A demethylation modification has been proven to promote CSC-associated All-Trans Retinoic Acid therapy resistance [138–140].

##### Adenosine-to-inosine (A-to-I) conversion

Adenosine-to-inosine (A-to-I) conversion has also become a widespread part of the regulatory networks of CSCs. Initially, A-to-I conversion alters adenosine in double-stranded RNAs into inosine, and then inosine is recognized as guanine at the molecular level. This process is catalyzed by the adenosine deaminase (ADAR) family, which includes ADAR1, ADAR2 and ADAR3 [141]. Of note, A-to-I conversion makes it easy for CSCs to generate novel binding sites for tumor-regulating factors and produce new proteins with tumor-regulating effects [142, 143]. According to different ADARs, we will introduce how A-to-I conversion regulates the sphere-formation and proliferation ability of CSCs.

ADAR1 is actively and functionally expressed in various cancer types, such as liver, colorectal and thyroid cancers. For instance, ADAR1 catalyzes the A-to-I conversion of antizyme inhibitor 1 (AZIN1) mRNA in CSCs from HCC [144] and CRC [145], which is crucial to maintain the sphere-formation ability of CSCs. Otherwise, Cyclin-dependent serine/threonine protein kinase 13 (CDK13) is required for constitutive and alternative pre-mRNA splicing in thyroid cancer. Julia Ramírez‑Moya et al. demonstrated that ADAR1 catalyzed the A-to-I conversion at CDK13 mRNA to enhance CSC proliferation, which provides an advantage for thyroid cancer progression [146]. A similar phenomenon was also observed in HCC [147].

ADAR2 also participates in A-to-I conversion control of mRNA in CSCs. However, ADAR2 plays both promotive and inhibitory roles. Glutamate receptor subunit B (GRIA2) competitively binds calcium transporters to block calcium transportation and excitatory synaptic transmission [148]. In glioblastoma, ADAR2-catalyzed A-to-I conversion activates GRIA2 mRNA to promote CSC proliferation [149]. Conversely, ADAR2-mediated blocking of some genes also regulates CSCs. Increasing evidence has proven that ADAR2 can block CSC proliferation by inactivating podocalyxin-like (PODXL) in gastric cancer and inhibiting COPA (coatomer protein complex, subunit α) in HCC [150, 151]. In contrast to ADAR1 and ADAR2, ADAR3 is exclusively expressed in the brain and is not catalytically active. However, ADAR3 also regulates CSC proliferation in glioblastoma by directly competing with ADAR2 at the editing site of GRIA2 to inhibit ADAR2 [149].

#### Noncoding RNA-mediated control of mRNAs

Coding RNAs, also called mRNAs, represent the single-stranded RNAs that store genetic information and can be translated into proteins [152]. The remaining RNAs are known as noncoding RNAs, and they function in cellular mechanisms and gene regulation [153]. Noncoding RNAs include miRNAs, lncRNAs, rRNAs, tRNAs, snRNAs and snoRNAs [154]. Since most studies of CSCs are related to miRNAs and lncRNAs, in this review, we will only introduce these two types of noncoding RNA-mediated control of mRNA.

##### MicroRNAs (miRNAs)

MiRNAs are small noncoding RNAs. More than 2,000 miRNAs have been shown to regulate gene expression by recognizing cognate sequences and participating in transcriptional processes [155, 156]. To date, miRNAs have been largely identified in the fields of development and disease regulation, particularly in cancers [157]. Increasing evidence has illustrated the connection between various miRNAs and cancers, and these miRNAs can be divided into two classes: tumor suppressor class and tumor-promoting class.

On the one hand, the let-7 family and miR34 are members of the tumor suppressor class of miRNAs, which act in suppressing CSC stemness, reversing therapy resistance and inhibiting CSC-associated tumor metastasis [158]. Fengyan Yu et al. suggested that let-7 silenced the mRNAs of several oncogenes in breast cancer and negatively regulated CSC stemness [159]. Additionally, let-7-modulated mRNA silencing even arrested the G2-M phase of the CSC cell cycle in prostate cancer [160]. In gastric and ovarian cancers, let-7-induced posttranscriptional modification not only inhibits CSC self-renewal but also reverses chemoresistance [161, 162]. In addition to the let-7 family, miR34-a also has tumor suppressor functions. In various cancer types, miR-34a targets mRNAs of tumor-promoting genes to inhibit CSC self-renewal and proliferation [163, 164]. Apart from these findings, data also showed that the miR34-mediated inhibition of mRNAs of EMT-related TFs contributed to the inhibition of CSC-associated tumor metastasis in head and neck squamous cell carcinoma (HNSCC) and prostate cancer [165, 166].

On the other hand, miRNAs also have tumor-promoting functions. miR21 and miR221 are involved in this class of miRNAs that promote CSC stemness maintenance, CSC-associated tumor metastasis and CSC-associated therapy resistance [158]. MiR-21 is one of the first onco-miRNAs found to be overexpressed in multiple human cancers [167–169]. In pancreatic cancer and glioma, the posttranscriptional regulation of CSC-related TFs by miR-21 is an important step in maintaining CSC stemness [167, 170]. In breast cancer, miR-21 facilitates CSC metastasis by upregulating mesenchymal markers or synergistically regulating HIF-1α mRNA [171]. MiR-221 is another onco-miRNA. Quaking gene 5 (QKI-5) is an isoform of the QKI gene that can suppress the sphere-formation ability of CSCs and tumor formation. QKI-5 is downregulated in CRC through miR-221-dependent modulation [172, 173]. In addition, it has been reported that miR-221 promotes CSC-associated gemcitabine resistance at the posttranscriptional level in pancreatic cancer [170].

##### Long noncoding RNAs (lncRNAs)

Long noncoding RNAs (lncRNAs) are a class of transcripts encoded by the genome that are not translated into proteins. LncRNAs play key roles in various physiological and pathological processes, such as chromatin dynamics regulation, RNA processing, protein translation and stabilization [174]. Notably, aberrant expression and mutation of lncRNAs exist in most cancer types and play an essential role in the posttranscriptional control of CSCs [175]. Recently, increasing research has focused on the lncRNA-related posttranscriptional control of mRNAs in maintaining CSC stemness and proliferation ability [176]. This control could be classified into two ways, direct control and indirect control, and will be described separately in the following section.

Direct control of lncRNAs on mRNAs means that lncRNAs bind to target mRNA sequences directly to modulate mRNA stabilization or splicing. In breast cancer, lncRNA ROPM stabilizes PLA2G16 mRNA by binding to the 3’UTR terminal, activating the PI3K, WNT/β-catenin and Hippo signaling pathways to maintain CSC stemness and helping with tumor development [177]. Indirect control of lncRNAs on mRNAs involves two mechanisms. On the one hand, lncRNAs bind to mRNAs through RBPs as mediators. For example, the lncRNA KB-1980E6.3 regulation of c-Myc mRNA requires IMP1 as a mediator for combination and then maintains CSC stemness in breast cancer by upregulating stemness-related TF expression [178, 179]. On the other hand, lncRNAs act as “sponges” for miRNAs in the regulation of CSC properties. Emerging evidence demonstrates that lncRNA MALAT1 inhibits miR-375, miR-200c and miR-145 to promote stemness-related mRNA expression and maintain CSC stemness in various solid cancers [180–183]. LncRNA LOCCS blocks the activities of miR-93 to maintain the proliferation ability of CSCs in colon cancer [184]. Additionally, the overexpression of another lncRNA CCAT2 of CSCs in colon cancer plays the same role by inhibiting miR-145 [185].

### Epigenetic modification control of CSCs

Epigenetic modifications mainly target genetic loci for transcriptional mechanisms and nucleosome remodeling without influencing the primary DNA sequence. DNA modification, histone modification and chromatin remodeling are three types of epigenetic modifications that maintain the stemness of CSCs [186]. Here, we only emphasize the functions of DNA modification and histone modification in this review (Fig. 1).

#### DNA modification

DNA methylation or demethylation is the most common DNA modification at the epigenetic level, and the modification site usually occurs at the guanine residues (CpG) [187]. DNA modification is crucial in cell development, apoptosis and differentiation [188]. In many cancer types, DNA methylation and demethylation change the expression level of some genes to regulate CSC characteristics [12].

Excessive DNA methylation can be achieved by the tumor itself and participate in CSC stemness maintenance and CSC-associated therapy resistance. For example, leucine-rich repeat containing G protein-coupled receptor (LGR5) is a well-known CSC marker in colon cancer [189]. DNA methylation-induced increased expression of LGR5 maintains the stemness of CSCs and promotes CSC-associated resistance to 5-fluorouracil [190]. Otherwise, gene mutations also lead to excessive DNA methylation of several enhancers at the epigenetic level. One study suggested that mutations in DNMT3 are related to aberrant stem cell gene expression and maintain CSC stemness in AML [191, 192].

Furthermore, DNA demethylation plays a role in regulating the sphere-formation ability of CSCs and CSC-associated therapy resistance. Hyun-Mi Kwon et al. demonstrated that DNMT1 inhibitors affect the sphere-formation ability of CSCs by effectively suppressing the expression of several TFs in pancreatic cancer, as well as in ovarian cancer and lung cancer [193–195]. In another study, DNMT1 inhibitor-mediated demethylation contributes to CSC resistance to sorafenib treatment [196].

#### Histone modification

Histone modification involves methylation, phosphorylation, acetylation, ubiquitination, adenylation and ADP ribosylation. Since most studies focus on the histone methylation control of CSCs, we only introduce this particular modification in the section. Histone methylation refers to the methylation of lysine (Lys, K) and arginine (Arg, R), which can be recognized by histone readers and initiate expression changes. Different amounts of methylation of histones at different sites may lead to gene activation or silencing [197].

The methylation of the Lys4 and Lys36 residues of histone H3 (H3K4me and H3K36me3) often results in targeted gene activation [198]. Increasing evidence has shown that the methylation of H3K4me can maintain CSC stemness and CSC-associated therapy resistance [198]. For example, scientists have found that the self-renewal characteristics of leukemia stem cells are maintained in a hyper-H3K4me3 state [199]. Several TFs genes are reported to be methylated at H3K4me in breast cancer, which maintains the stemness of CSCs [200]. In addition, H3K4me3 increased at the promoters of several stemness TFs and markers, which account for CSC self-renewal and proliferation in CRC [201]. Regarding CSC-associated therapy resistance, researches have demonstrated that GALNT10 facilitates CSC-associated platin resistance in ovarian cancer treatment through epigenetic modification in an H3K4me-dependent methylation manner [202, 203]. In addition, Qinghai Lin et al. indicated that H3K36me3-dependent histone modification of Oct4, Sox2 and Nanog at the promoter region is critical in maintaining CSC stemness in HCC [204].

Conversely, the methylation of H3K9me2 and H3K27me3 is generally associated with gene transcription repression, which also participates in CSC stemness maintenance [197]. In glioblastoma, H3K9me2 modification of CD133 and Sox2 is important in regulating CSC self-renewal [205]. Moreover, H3K27me3 modification promotes CSC self-renewal and differentiation in both EZH2 dependent and independent ways, thereby resulting in tumorigenesis in glioblastoma, ovarian cancer and prostate cancer [206–208].

### Tumor microenvironment (TME) control of CSCs

The regulation of the tumor microenvironment (TME) is also one of the most important mechanisms within the regulatory networks of CSCs. The TME is the immune environment that affects tumor growth and metastasis due to the continuous interaction between tumor cells, nontumor cells (such as stromal cells, immune cells, endothelial cells, etc.) and noncellular factors (such as metabolism, etc.) [209]. Among the nontumor cells of the TME, cancer-associated fibroblasts (CAFs) are the most representative stromal cells, and tumor-associated macrophages (TAMs) are the most essential immune cells that control CSCs and promote tumor progression. Apart from that, metabolism is intricately linked to the TME, which was also related to CSCs characteristics maintenance. Hence, in this section, we mainly introduce the control of CSCs by CAFs, TAMs and metabolic factors (Fig. 2).

#### Cancer-associated fibroblasts (CAFs)

In the TME, CAFs are the most abundant stromal components, and other cells include undifferentiated mesenchymal stem cells (MSCs), endothelial cells and pericytes [210]. CAFs maintain CSC stemness, sphere-formation ability, CSC-associated tumor metastasis and CSC-associated therapy resistance by shaping the extracellular matrix [211].

For stemness maintenance, emerging evidence has demonstrated that CAF-induced STAT3 signaling activation leads to hepatocyte growth factor (HGF) and IL-6 over-secretion to enhance CD24 expression on CSCs in HCC [212]. Tsuyada A et al. indicated that in breast cancer, CAFs secrete CCL2 to promote tumor progression by maintaining the stemness and sphere-formation ability of CSCs [213]. In addition, CAFs also play a critical role in maintaining CSC-associated tumor metastasis via CAF-derived cytokine secretion and TF expression [214, 215]. Furthermore, CSC-associated chemoresistance can be realized by the CAFs-provided supporting tumor niche enriched with IL-6 and IL-8 in clinical samples of breast and lung cancers [216]. Furthermore, CSC-associated chemoresistance can also be achieved through CAF-induced signaling pathway activation in breast cancer and CRC, such as the TGF pathway and β-catenin pathway [217–219].

#### Tumor-associated macrophages (TAMs)

Generally, tumor-infiltrating immune cells include neutrophils, lymphocytes, monocytes, macrophages and their immature precursors [220]. It has been reported that macrophages have two phenotypes, M1 and M2, which are heterogenic [221]. The M1 phenotype triggers proinflammatory factors to activate antitumor properties, whereas M2 macrophages are tumor-associated macrophages (TAMs) that secrete chemokines and ligands to achieve the same purpose. Meanwhile, TAMs can specifically promote tumor growth by maintaining CSC stemness and proliferation [222].

For example, TAMs possibly influenced the binding between hyaluronic acid (HA) and CD44, thus maintaining CSC stemness via the PI3K/4EBP1/Sox2 pathway in HNSCC [223]. In pancreatic cancer, the stemness of CSCs is closely associated with TAM-secreted interferon-stimulated gene 15 (ISG15), a protein factor with immunomodulatory properties [224]. Moreover, TAMs physically interact with CSCs from breast cancer through EphA4/ezrin and CD90/CD11b to maintain the stemness of CSCs [225]. IL-6 secreted by TAMs plays an important role in the expression of CD44 and the proliferation of CSC, while blocking the IL6 receptor reverses this process [226]. Additionally, CAFs recruit TAMs in a CXCL12/CXCR4-dependent manner, which orchestrates EMT and CSC stemness in oral squamous cell carcinoma [227].

#### Metabolic controls of CSCs in TME

In addition to the regulation of CSCs by CAFs and TAMs, metabolic control also plays a crucial role in maintaining CSC characteristics within the TME [228]. Glycolysis, glutaminolysis, and lipogenesis are three significant metabolic characteristics of CSCs. First and foremost, tumor cells primarily rely on glucose as their primary fuel source, especially CSCs [229]. Upregulated glycolysis has been closely associated with CSC metabolism in breast cancer and glioblastoma [230, 231]. Furthermore, CSCs often exhibit a higher demand for glutamine, making glutaminolysis an essential factor in regulating CSCs [232]. Glutamine is known to be involved in nucleotide and amino acid biosynthesis in CSCs of neuroblastoma [233]. Additionally, in the metabolic regulation of HCC and CRC, glutaminolysis has been reported to play a role in maintaining CSC characteristics through demethylation and DNA damage repair [234, 235]. Furthermore, lipogenesis is another critical metabolic characteristic of CSCs [236]. Growing evidence suggests that upregulated lipogenesis is observed in CSCs derived from pancreatic cancer [237]. Additionally, scientists have discovered that increased fatty acid oxidation is crucial for maintaining CSCs in breast cancer [238] and leukemic cells [239].

Notably, hypoxia-inducible factor (HIF) is a critical factor in the TME that can influence these three metabolic characteristics of CSCs [240]. HIF has been shown to mediate a switch from oxidative to glycolytic metabolism in CSCs under hypoxic conditions, providing protection against oxidative damage in breast cancer [241]. In HCC, hypoxia was demonstrated to enhance the self-renewal ability of CSCs in an HIF-1α-dependent and HIF-2α-dependent manner [242]. Consequently, emerging research has identified metabolic agents as potential therapeutic agents for targeting CSCs, which will be discussed in detail in the following section.

### EMT control of CSCs

Epithelial-mesenchymal transformation (EMT) is a dedifferentiation process that converts polarized epithelial cells into cells with a mesenchymal phenotype, which occurs by losing adhesion with neighboring cells [243]. The transition from the epithelial phenotype to the mesenchymal phenotype bestows cells with multiple capabilities, including stem cell properties [7]. The EMT process often occurs at the early stage of embryonic development; however, it is also linked to several pathological processes, such as cancers process [244]. Moreover, loss of E-cadherin is considered as the hallmark of EMT [245]. Increasing evidence suggests that EMT-inducing transcription factors (EMT-TFs), Snail, Slug, ZEB1 and Twist, downregulate E-cadherin expression and further promote the EMT process, which results in cancer cells losing epithelial properties but acquiring mesenchymal properties. And this process could promote stem markers expression, which could be the basic that EMT process maintains CSC stemness, CSC-associated tumor metastasis and CSC-associated therapy resistance [246].

Firstly, Snail is the most significant EMT-TF that plays a role in CSC stemness maintenance and CSC-associated tumor metastasis [245]. For CSC stemness maintenance, Sendurai A. Mani et al. demonstrated that in breast cancer, Snail-induced EMT process was responsible for the generation of CSC through the loss of E-cadherin expression. In addition, the transformed CSC acquires high CD44 expression to maintain stemness characteristics [247–249]. Apart from that, Slug can evoke similar functions as Snail and maintain CSC stemness in cancer [245]. In glioblastoma, overexpression of Slug induces cancer cells to lose E-cadherin then undergo EMT process, which switch cancer cells to CSC with self-renewal property and maintain CSC stemness through mesenchymal transformation-induced stem markers expression [250]. In addition, ZEB1-mediated EMT is also involved in maintaining CSC stemness [245]. Accumulating evidence in pancreatic cancer has demonstrated that ZEB1 regulates the EMT process, driving the transformation of cancer cells into CSCs with self-renewal properties [251]. Finally, Twist1 primes epithelial cells for stemness characteristics and maintains CSC stemness through EMT [245]. Novel research in HNSCC has revealed that the overexpression of Twist1 is essential for suppressing E-cadherin expression in cancer cells, inducing EMT, and thereby imparting cancer epithelial cells with stem cell properties, which help maintain CSC stemness [252]. Apart from CSC stemness maintenance, Twist also plays a role in CSC-associated therapy resistance. For example, it has been observed in colon cancer that E-cadherin downregulation induced by Twist promotes EMT process, which contributes to CSC phenotype transformation and is critical for CSC-associated irinotecan resistance [253].

However, CSC stemness does not parallel the degree of EMT, which means that extreme EMT leads to cells exhibiting a fully differentiated state rather than the stem-like phenotype [254, 255].

## Regulation of signaling pathways in CSCs

**Fig. 3:**
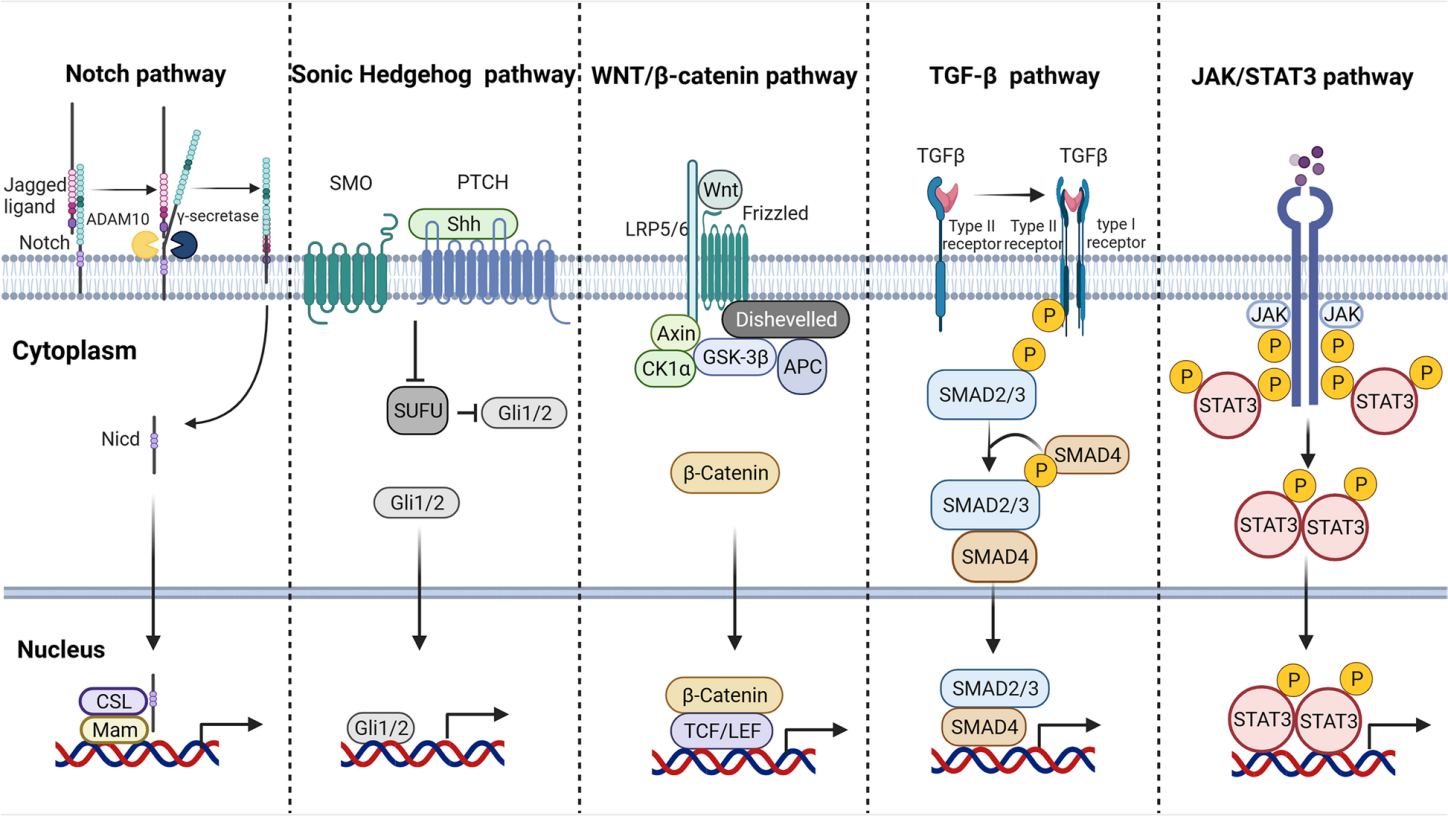
The signaling pathways controlling CSCs. Several signaling pathways play critical roles in malignancy transformation and tumor development, particularly within the CSC regulatory network. The accompanying figure introduces the five primary signaling pathways and outlines the mechanisms of signal transmission within each pathway. Notably, in the Notch signaling pathway, the core component, NICD, undergoes three cleavage events before entering the nucleus to promote gene transcription. Shh signaling can be activated in CSCs by inhibiting SMO-mediated Gli cleavage. WNT/β-catenin signaling is facilitated by the accumulation of inactive β-catenin, regulated by GSK-3β, and requires the involvement of Axin and Dishevelled. TGF-β recruits its receptors and initiates phosphorylation through serine/threonine kinase domains, subsequently translocating phosphorylated SMAD into the nucleus. In the JAK/STAT3 signaling pathway, signals are transmitted through transphosphorylation events downstream

Evidence suggests that many signaling pathways are involved in the regulatory networks of CSCs. Instead of relying on a single regulator, these processes are governed by intricate interwoven networks of signaling pathways, as depicted in Fig. 3. The networks include the Notch, WNT/β-catenin, Sonic hedgehog (Shh), TGF-β and JAK/STAT3 signaling pathways. Therefore, this section describes how these signaling pathways contribute to the maintenance of CSC characteristics, CSC-associated tumor metastasis and CSC-associated therapy resistance.

### Notch signaling

The Notch signaling pathway plays a crucial role in regulating cancer progression across various tumor types, with the Notch Intracellular Domain (NICD) serving as one of its key effectors. Following three sequential cleavage events mediated by furin, ADAM, and γ-secretase, Notch ICD translocates into the nucleus, where it functions as a TF [256]. However, Notch signaling can play either oncogenic or suppressive functions depending on specific condition [257]. Increasing evidence has demonstrated that aberrant Notch activation in CSCs is beneficial in stemness maintenance and CSC-associated tumor metastasis [258].

For instance, in breast cancer and pancreatic cancer, Notch signaling, which is activated by Sydecan-1 and ZEB, respectively, contributed to the maintenance of CSCs stemness [259, 260]. In addition, the Notch signaling pathway is activated by HIF-1α, which could promote CSC-associated tumor metastasis in lung cancer, ovarian cancer and breast cancer [261–264]. Contrary, it has been found that CSCs could regulate Notch signaling in turn. A few studies showed that many genes expressed by CSCs could activate Notch signaling. For instance, it has been demonstrated that overexpression of HIST2H2BF and STRAP in CSCs significantly contributes to the activation of Notch signaling and the release of NICD [265, 266].

### WNT/β-catenin signaling

Classical WNT/β-catenin pathway requires the binding of WNT ligands to Frizzled and LRP receptors, which rescues the inhibition of β-catenin by APC, axin and GSK-3β [267]. The WNT/β-catenin signaling pathway has been found involved in many biological processes in decades. Meanwhile, the relationship between WNT/β-catenin signaling pathway and pathological processes, especially cancer development, has also been gradually revealed [268, 269]. In the study of the underlying mechanism, scientists have discovered that the WNT/β-catenin signaling pathway could be one of the key cascades in the regulation of CSCs [270]. Notably, hyperactivated WNT/β-catenin signaling in the CSC population is responsible for maintaining CSC stemness, promoting the sphere-forming ability of CSCs, and contributing to therapy resistance associated with CSCs [271].

For example, in colon cancer, p53 and myofibroblasts are critical in activating and maintaining CSC stemness through WNT/β-catenin signaling modulation [272, 273]. Moreover, WNT/β-catenin-dependent CD44 expression is positively correlated with CSC stemness in melanoma and breast cancer [274–276]. Additionally, TCF7, a member of the TCF/LEF family, is a downstream target of WNT and is essential for maintaining CSC stemness in pancreatic cancer [277]. In addition, scientists have identified that the activated WNT/β-catenin signaling is critical for both sphere-formation ability and CSC-associated chemo-/radio-resistance in gastric cancer [278–280]. The same phenomenon is found in CRC induced by overexpression of CD45 [281].

### Sonic hedgehog (Shh) signaling

The Sonic Hedgehog (Shh) signaling networks include extracellular hedgehog ligands, the transmembrane protein receptor PTCH, the transmembrane protein SMO, intermediate transduction molecules and the downstream molecule Gli [282]. In the presence of Shh, SMO inhibition by PTCH is relieved, and full-length Gli activates different target genes for further transcription processes [283]. Similar to other signaling pathways, Shh signaling is also involved in several cancers, such as colorectal, breast and lung cancers [284]. Data have shown that Shh signaling is another key pathway in regulating CSC characteristics [270].

In thyroid tumors, Shh signaling regulates CSC stemness through Gli expression-mediated Bmi1 and Sox2 expression at the posttranscriptional and transcriptional levels [285]. In addition, increasing evidence has proven that several upstream regulators of the Shh-SMO-Gli axis are important in maintaining CSC stemness. For example, in CD138^+^ myeloma stem cells, Shh signaling is activated by RARα2 [286] Referring to the contribution of Shh signaling to CSC-associated tumor metastasis, the study has shown that the degradation of Gli by RUNX3-mediated ubiquitination could reduce CSC-associated tumor metastasis in CRC [287].

### TGF- signaling

TGF-β is involved in multiple cellular processes, such as cell proliferation, development and homeostasis [288]. Mechanically, the TGF-β/type II receptor complex recruits the type I receptor and undergoes a phosphorylation event, followed by the recruitment and phosphorylation of the SMAD family that regulates downstream gene expression [289]. At the pathological level, it is a fundamental promoter of CSC self-renewal maintenance and CSC-associated tumor metastasis [290].

Data have illustrated that the TGF-β expression level is positively correlated with the CD44^+^ CSC population in breast and gastric cancers, which shows powerful self-renewal ability [291–293]. Moreover, Kim BN et al. demonstrated that TGF-β-mediated DNA demethylation of Slug and stemness-related TFs can promote CSC self-renewal ability [294, 295]. Apart from maintaining the self-renewal of CSCs, Yeh HW et al. emphasized the function of TGF-β signaling in regulating EMT and CSC-associated tumor metastasis in both liver and lung cancers by increasing Snail expression [296]. Moreover, TGF-β signaling can regulate CSC-associated tumor metastasis at the posttranscriptional modification level. In pancreatic cancer, TGF-β/SMAD signaling regulates CSCs by inducing miR-100 and miR-125b but blocking let-7a [297].

### JAK/STAT3 signaling

The JAK/STAT3 signaling pathway participates in many physiological processes, including cell proliferation, immune regulation and differentiation [298]. The tyrosine kinase-related receptors, JAK and STAT3 are three main components within the signaling pathway. Cytokines and growth factors such as interferon, interleukin, EGF and PDGF transmit signals depending on this pathway. In most human cancers, JAK/STAT3 signaling is involved in CSC stemness maintenance and CSC-associated tumor metastasis [299].

Reports have illustrated that JAK2/STAT3 signaling upregulates cyclin D2 and stemness-related TFs to persistently maintain CSC stemness in cancers [300–302]. Moreover, a novel report showed that the critical role of oncostatin M in CSC stemness maintenance is realized through the JAK/STAT3 signaling pathway [303, 304]. Alternatively, data have revealed that inhibition of the JAK2/STAT3 signaling pathway results in the downregulation of CSC markers in cancers, which weakens the stemness characteristics of CSCs [305–307]. In addition to maintaining the stemness of CSCs, JAK/STAT3 signaling also leads to CSC-associated tumor metastasis by regulating the EMT process. It has been observed that the positive feedback autocrine loop between osteopontin and the JAK/STAT3 pathway results in the EMT process, which participates in the persistent enhancement of CSC-associated tumor metastasis [308, 309].

## CSCs in cancer therapy resistance

**Fig. 4:**
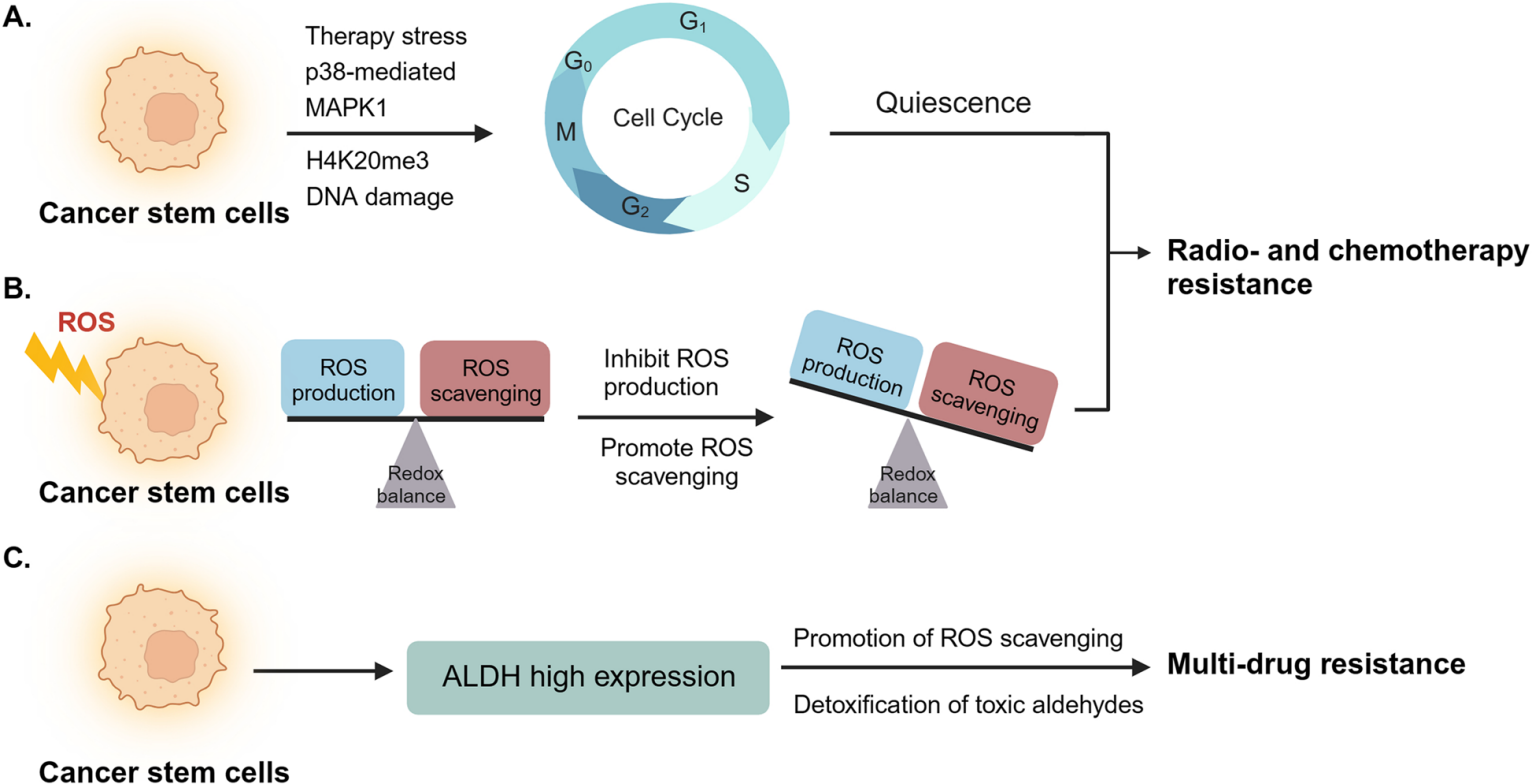
CSCs in cancer therapy resistance. CSCs demonstrate greater resistance to cancer therapy compared to regular cancer cells, making them more likely to evade radiotherapy and chemotherapy and increasing the risk of tumor relapse. Several factors contribute to the development of therapy resistance in CSCs. **A** CSCs can enter a quiescent state when exposed to environmental stress, such as therapy-induced stress, specific molecular stress, and DNA damage stress, enabling them to acquire resistance to radiotherapy and chemotherapy. **B** The disruption in the balance between ROS production and ROS scavenging in CSCs leads to CSC-related resistance to radiotherapy and chemotherapy. **C** High ALDH expression in CSCs enhances ROS scavenging and detoxification of toxic aldehydes, leading to multidrug resistance

In the last few decades, multiple therapeutic strategies have been applied in the treatment of cancer. These strategies fall into three categories: surgery, chemotherapy and radiotherapy [3, 310]. In clinical practice, these treatment strategies often face challenges, with one significant obstacle being CSCs-associated therapy resistance [311]. The CSC population consistently promotes a dynamic phenotypic switch between stem and non-stem states to resist cancer therapies [312]. Several factors can be utilized by CSCs to induce cancer therapy resistance: quiescence, reactive oxygen species (ROS) and aldehyde dehydrogenase (ALDH) (Fig. 4).

### Quiescence-mediated radiation and chemotherapeutic resistance

Quiescence is a cellular state observed in stem cells, wherein these cells remain in the nondividing G0 phase. Stem cells in this state could escape from stress and then switch their phenotype to increase the proliferation ability after the stress is over. For instance, CSCs remain quiescent in response to hypoxia, nutritional deprivation and other stresses, but can reawaken in favorable conditions, leading to tumor relapse [313]. Since most agents target the proliferated state of cancer cells, CSCs could take advantage of the quiescent state as a mechanism of therapy resistance, resulting in tumor relapse [314].

For instance, it has been reported that a slow-cycling quiescent CSC population can evade chemotherapy in cases of melanoma and chronic myeloid leukemia (CML), potentially leading to tumor relapse [315, 316]. Other reports have shown that p38-mediated MAPK1 activation or H4K20me3 methylation-dependent formation of tighter heterochromatin can lead to CSC quiescence, impacting therapy resistance in cancers [317, 318]. Moreover, the DNA damage response usually induces cell cycle arrest and cell death. In esophageal cancer and glioma, quiescent CSCs exhibit resistance to DNA damage induced by radiotherapy or chemotherapy [319, 320]. Additionally, in bladder cancer, it has been reported that quiescent CSCs re-enter the cell division cycle in response to damage caused by gemcitabine and cisplatin [321].

### ROS-mediated radiation and chemotherapeutic resistance

Reactive oxygen species (ROS) are oxygen-containing molecules characterized by their short-lived and highly reactive properties. The production of ROS is linked to cellular physiological processes, including respiration, energy consumption, and enzyme activities [322]. ROS are typically regarded as harmful metabolites that can be involved in causing DNA damage and triggering the DNA damage response [323]. Compared with cancer cells, CSCs are much more responsive to variations in oxygen levels. Moreover, studies have demonstrated how CSCs use ROS to resist radio- and chemotherapy-derived oxidative stress [324].

Data have shown that CSCs from ovarian cancer led to an increase in Nrf2 levels [325], CSCs from HCC over-activate Prx2 expression [326], and CSCs from pancreatic cancer upregulate glycolysis-induced DCLK1 expression, all of which enhance ROS scavenging and lead to chemoresistance [327]. Moreover, CSCs exhibit radio-resistance properties not only by reducing ROS levels but also by enhancing ROS defenses in breast and some brain cancers [328]. These studies indicate that disruption of the redox balance in CSCs is a possible and promising strategy in cancer treatment.

### ALDH-mediated multidrug resistance

Multidrug resistance (MDR) is a special kind of chemoresistance in which cancer cells resist chemotherapeutic agents with different structures and mechanisms of action. Aldehyde dehydrogenase (ALDH) is an enzyme with functions related to aldehyde detoxification and retinoic acid synthesis, playing vital roles in cellular mechanisms. It is also considered a potential selective marker for CSCs in various cancer types [329]. Currently, it is regarded as a target for CSC-associated multi-agent resistance.

At first, the CSC-associated multidrug resistance is positively correlated with ALDH expression level [330]. For instance, in various cancers, the ALDH1^+^ CSC population demonstrates resistance to chemotherapy [331]. ALDH can mediate multidrug resistance through the following two mechanisms. Firstly, it impacts ROS levels. Studies have revealed that ALDH helps maintain ROS levels low enough to prevent apoptosis induced by therapeutic agents in lung cancer CSCs [332]. Secondly, it facilitates the detoxification of toxic aldehydes. In gynecologic malignancies, ALDH in CSCs detoxifies toxic aldehydes into less harmful carboxy compounds, contributing to CSC-associated therapy resistance [333].

## CSC-associated targeting agents for cancer

As mentioned above, cell surface markers, multiple regulatory networks and signaling pathways have tight connection with the modulation of CSC characteristics. Targeting these mechanisms is of utmost importance to eradicate both CSCs and the bulk tumor population. In this review, CSC-associated targeting agents are classified into five subgroups: agents targeting cell surface markers of CSCs, agents targeting transcriptional factors of CSCs, agents targeting the metabolism of CSCs, agents targeting CSC-associated signaling pathways and agents targeting epigenetic modifications. The summary of agents targeting CSCs in clinical and preclinical studies is presented in Table 1.

**Table 1:** Summary of the agents targeting for CSCs in clinical and preclinical

**Agent**	**Target**	**Condition**	**Phase**	**Sample size**	**NCT/Reference**	**Status**
**Surface Marker Inhibitors**						
Bivatuzumab mertansine	CD44v6	Breast Cancer	I	24 participants	NCT02254005	Completed
		Head and Neck Cancer	I	31 participants	NCT02254018	Completed
Catumaxomab	EpCAM/CD3	Bladder Cancer	I	30 participants	NCT04819399	Recruiting
		Ovarian Cancer	II	47 participants	NCT00377429	Completed
		Colon Cancer, Breast Cancer	II	258 participants	NCT00836654	Completed
		Gastric Cancer	II	70 participants	NCT00464893	Completed
		Stomach Cancer	III	282 participants	NCT04222114	Recruiting
CC-90002	CD47	Hematologic Cancer	I	60 participants	NCT02367196	Completed
CSL362	CD123	AML	I	30 participants	NCT01632852	Completed
Talacotuzumab (formerly CSL362)	CD123	AML	III	326 participants	NCT02472145	Completed
Magrolimab (Hu5F9-G4)	CD47	Solid Tumors	I	88 participants	NCT02216409	Completed
		Ovarian Cancer	I	34 participants	NCT03558139	Completed
		AML	I	20 participants	NCT02678338	Completed
		Large B Cell Lymphoma	I	30 participants	NCT03527147	Completed
		Bladder Cancer	II	645 participants	NCT03869190	Recruiting
IBI188	CD47	Advanced Malignancies	I	20 participants	NCT03763149	Completed
		Solid Tumors	I	120 participants	NCT04861948	Recruiting
		Acute Myeloid Leukemia	I	126 participants	NCT04485052	Recruiting
IMGN632	CD123	Blastic Plasmacytoid DC Neoplasm	I	252 participants	NCT03386513	Recruiting
		AML	II	274 participants	NCT04086264	Recruiting
IMGN779	CD33	AML	I	62 participants	NCT02674763	Completed
JNJ-63709178	CD123/CD3	AML	I	62 participants	NCT02715011	Completed
Flotetuzumab(MGD006)	CD123	Myeloid Leukemia	II	25 participants	NCT04582864	Recruiting
		Hematologic Cancer	I	40 participants	NCT04681105	Recruiting
Gemtuzumab Ozogamicin	CD33	AML	I	24 participants	NCT04070768	Recruiting
		Blood Cancers	II	100 participants	NCT03589729	Recruiting
		AML	III	168 participants	NCT00962767	Completed
RO5429083	CD44	Solid Tumors	I	65 participants	NCT01358903	Completed
		AML	I	44 participants	NCT01641250	Completed
SGN-CD123A	CD123	AML	I	17 participants	NCT02848248	Terminated
SRF231	CD47	Solid Tumors Hematologic Neoplasmas	I	148 participants	NCT03512340	Completed
Tagraxofusp (SL-401)	IL3R-targeting Fusion Protein	Dendritic Cell Neoplasm	II	40 participants	NCT04216524	Recruiting
	CD123	Acute Myeloid Leukemia	II	50 participants	NCT04342962	Recruiting
		CML	II	130 participants	NCT02268253	Recruiting
Talacotuzumab	CD123	AML	III	326 participants	NCT02472145	Completed
TTI-621	CD47	Hematologic Cancer	I	260 participants	NCT02663518	Recruiting
		Multiple Myeloma	I	40 participants	NCT05139225	Recruiting
		Leiomyosarcoma	I	80 participants	NCT04996004	Recruiting
Vadastuximab Talirine (SGN-CD33A)	CD33	AML	I	116 participants	NCT02326584	Completed
XmAb14045	CD123	Hematologic Cancer	I	120 participants	NCT02730312	Completed
4SCAR-T	CD22/CD123/CD38/ CD10/CD20	CD19 Negative B-cell Malignancies	I II	100 participants	NCT04430530	Recruiting
Anti-CD33 CAR NK cells	CD33	AML	I	27 participants	NCT05008575	Recruiting
4SCAR T cells	CD44v6	Breast Cancer	I II	100 participants	NCT04430595	Recruiting
**Transcriptional factor inhibitors**						
Ivermectin	Oct4/Sox2/Nanog	Breast Cancer			[334]	Preclinical
		Solid Tumors			[335]	Preclinical
ZF-5985KD	Sox2	Breast Cancer			[336]	Preclinical
Peptide aptamer P42	Sox2	Esophageal squamous cell Carcinoma			[337]	Preclinical
Simvastatin	KLF4	Osteosarcoma			[338]	Preclinical
D347-2761	c-Myc	Osteosarcoma			[339]	Preclinical
T417	PBX1	Ovarian Cancer			[340]	Preclinical
**Metabolism inhibitors**						
2-deoxy-D-glucose	Glycolysis	Solid Tumors	I	50 participants	NCT00096707	Completed
		Prostate Cancer	I II	12 participants	NCT00633087	Terminated
CB-839	Glutaminolysis	Solid Tumors	I	210 participants	NCT02071862	Completed
		Hematological Tumors	I	25 participants	NCT02071888	Completed
TVB-2640	Lipogenesis	Breast Cancer	II	80 participants	NCT03179904	Recruiting
Omeprazole	Lipogenesis	Breast Cancer	II	42 participants	NCT02595372	Completed
PT2385	HIF	Renal cell Carcinoma	I	80 participants	NCT04989959	Recruiting
		Glioblastoma	II	24 participants	NCT03216499	Completed
Metformin	Glycolysis	Hepatocellular Carcinoma			[341]	Preclinical
		Prostate Cancer			[342]	Preclinical
Epigallocathechine gallate	Glycolysis	Pancreatic Cancer			[343]	Preclinical
R-HepG2	Glutaminolysis	Hepatocellular Carcinoma			[234]	Preclinical
alpha-ketoglutarate	Glutaminolysis	Colorectal Carcinoma			[235]	Preclinical
GSK864	Glutaminolysis	Glioblastoma			[344]	Preclinical
PT2399	HIF	Renal cell Carcinoma			[345]	Preclinical
32-134D	HIF	Hepatocellular Carcinoma			[346]	Preclinical
**Shh inhibitor**						
BMS-833923 (XL139)	SMO	Solid Tumor	I	12 participants	NCT01413906	Completed
		Basal Cell Carcinoma	I	53 participants	NCT00670189	Completed
		Stomach/Esophageal Cancer	I	39 participants	NCT00909402	Completed
		Small Cell Lung Carcinoma	I	5 participants	NCT00927875	Completed
		Leukemia	I II	33 participants	NCT01218477	Completed
Glasdegib	SMO	AML	III	730 participants	NCT03416179	Completed
		Soft Tissue Sarcoma	III	960 participants	NCT03784014	Recruiting
LDE225	SMO	Prostate Cancer	I	14 participants	NCT02111187	Completed
		Ovarian Cancer	I	15 participants	NCT02195973	Completed
		Solid Tumors	I	30 participants	NCT01954355	Completed
		Pancreatic Cancer	I	18 participants	NCT01487785	Completed
		Myeloid Malignancies	I	63 participants	NCT02129101	Completed
		Medulloblastoma	II	22 participants	NCT01708174	Completed
Vismodegib	Hedgehog	Solid Tumors	I	52 participants	NCT01209143	Completed
		Colorectal Cancer	II	199 participants	NCT00636610	Completed
Vismodegib	Hedgehog	Ovarian Cancer	II	104 participants	NCT00739661	Completed
		Pancreatic Cancer	II	118 participants	NCT01064622	Completed
		Gastric Cancer	II	124 participants	NCT00982592	Completed
		Basal Cell Carcinoma	IV	30 participants	NCT03610022	Recruiting
**Notch inhibitors**						
AL101	γ secretase	Adenoid Cystic Carcinoma	I	12 participants	NCT04973683	Recruiting
		Breast Cancer	II	67 participants	NCT04461600	Recruiting
BMS-906024	NOTCH	Solid Tumors	I	94 participants	NCT01292655	Completed
		Lymphoblastic Leukemia, Acute T-cell	I	31 participants	NCT01363817	Completed
CB-103	NOTCH	Solid Tumours/Haematological Malignancies	I II	200 participants	NCT03422679	Recruiting
		Breast Cancer	II	80 participants	NCT04714619	Active, not recruiting
LY3039478	γ secretase	Solid Tumor	I	94 participants	NCT02784795	Completed
		Plasma Cell Myeloma	I	18 participants	NCT03502577	Suspended
		T-cell Lymphoblastic Lymphoma	I	36 participants	NCT02518113	Completed
MK-0752	γ secretase	Pancreatic Cancer	I	44 participants	NCT01098344	Completed
		Breast Cancer	IV	22 participants	NCT00756717	Completed
RO4929097	γ secretase	Sarcoma	I II	78 participants	NCT01154452	Completed
RO4929097	γ secretase	Colorectal cancer	II	37 participants	NCT01116687	Completed
		Pancreatic Cancer	II	18 participants	NCT01232829	Completed
		NSCL	II	7 participants	NCT01070927	Completed
Brontictuzumab(OMP-52M51)	NOTCH	Colorectal Cancer	I	7 participants	NCT03031691	Completed
		Lymphoid Malignancies	I	24 participants	NCT01703572	Completed
		Solid Tumors	I	48 participants	NCT01778439	Completed
Demcizumab (OMP-21M18)	NOTCH 1	Solid Tumors	I	29 participants	NCT02722954	Completed
		NSCL	I	50 participants	NCT01189968	Completed
		Pancreatic Cancer	II	207 participants	NCT02289898	Completed
ABL001	DLL4	Solid Tumors	I	45 participants	NCT03292783	Completed
**WNT signaling**						
CWP232291	β-catenin	Multiple Myeloma	I	25 participants	NCT02426723	Completed
CWP232291	β-catenin	AML	I	69 participants	NCT01398462	Completed
PRI-724	CBP/β-catenin	Pancreatic Cancer	I	20 participants	NCT01764477	Completed
		Myeloid Malignancies	I II	49 participants	NCT01606579	Completed
OMP-54F28	Frizzled8	Liver Cancer	I	10 participants	NCT02069145	Completed
		Pancreatic Cancer	I	26 participants	NCT02050178	Completed
		Ovarian Cancer	I	37 participants	NCT02092363	Completed
OMP-54F28	Frizzled8	Solid Tumors	I	26 participants	NCT01608867	Completed
Vantictumab (OMP-18R5)	Frizzled7	Pancreatic Cancer	I	30 participants	NCT02005315	Completed
		Breast Cancer	I	37 participants	NCT01973309	Completed
		Solid Tumors	I	35 participants	NCT01345201	Completed
ETC-1922159	Porcupine	Solid Tumors	I	89 participants	NCT02521844	Recruiting
LGK974	Porcupine	Solid Tumors	I	185 participants	NCT01351103	Recruiting
		Colorectal Cancer	I II	20 participants	NCT02278133	Completed
**TGF-β inhibitors**						
AVID200	TGFβ	Solid Tumor	I	19 participants	NCT03834662	Active, not recruiting
LY3200882	TGFβRI	Solid Tumor	I	223 participants	NCT02937272	Active, not recruiting
Fresolimumab (GC1008)	TGF-β	Renal Cell Carcinoma/Melanoma	I	29 participants	NCT00356460	Completed
		NSCL	I II	24 participants	NCT02581787	Completed
		Breast Cancer	II	23 participants	NCT01401062	Completed
		Brain Tumors	II	12 participants	NCT01472731	Completed
		Mesothelioma	II	14 participants	NCT01112293	Completed
Galunisertib	Growth Factor-β Receptor I Kinase	Pancreatic Cancer	I	37 participants	NCT02734160	Completed
		Solid Tumor	I II	41 participants	NCT02423343	Completed
		Glioma	I II	75 participants	NCT01220271	Completed
		Hepatocellular Carcinoma	II	204 participants	NCT01246986	Completed
NIS793	TGF-β	Solid Tumor	I	120 participants	NCT02947165	Completed
		Colorectal Cancer	II	266 participants	NCT04952753	Recruiting
		Pancreatic Ductal Adenocarcinoma	III	490 participants	NCT04935359	Recruiting
Vactosertib (TEW-7197)	TGF-β	Solid Tumor	I	35 participants	NCT02160106	Completed
		Multiple Myeloma	I	18 participants	NCT03143985	Recruiting
		Desmoid Tumor	I II	24 participants	NCT03802084	Recruiting
		NSCL	II	55 participants	NCT04515979	Completed
**JAK inhibitors**						
AZD4205	Janus Kinase	NSCL	I II	10 participants	NCT03450330	Completed
		T Cell Lymphoma	II	160 participants	NCT04105010	Recruiting
SAR302503	JAK2 V617F	Solid Tumor	I	60 participants	NCT01836705	Completed
		Hematopoietic Neoplasm	II	97 participants	NCT01523171	Completed
Ruxolitinib	JAK	Breast Cancer	II	29 participants	NCT01594216	Completed
		AML	III	1000 participants	NCT03117751	Recruiting
SB1518	FLT3	Lymphoma	I	35 participants	NCT00741871	Completed
		AML	I	13 participants	NCT02323607	Completed
**DNMT inhibitors**						
Azacitidine	DNMT	AML	III	488 participants	NCT01074047	Completed
Decitabine	DNMT	Thyroid Cancer	II	12 participants	NCT00085293	Completed
		Solid Tumor	III	200 participants	NCT04292769	Recruiting
Decitabine	DNMT	Ovarian cancer	II III	500 participants	NCT02159820	Recruiting
		Hodgkin Lymphoma	II III	100 participants	NCT04510610	Recruiting
Guadecitabine (SGI-110)	DNMT	Colorectal Cancer	I	18 participants	NCT01966289	Completed
		MDS	I II	401 participants	NCT01261312	Completed
		Ovarian Cancer	II	120 participants	NCT01696032	Completed
		Hepatocellular Carcinoma	II	52 participants	NCT01752933	Completed
		AML	III	302 participants	NCT02920008	Completed
Disulfiram	DNMT1	Prostate Cancer	N/A	19 participants	NCT01118741	Completed
Aza-TdC	DNMT1	Solid Tumor	I	50 participants	NCT03366116	Recruiting
NTX-301	DNMT	Myeloid Malignancies	I	20 participants	NCT04167917	Recruiting
**HDAC inhibitors**						
Belinostat	Class I/II HDACs	Solid Tumors/Hematological Malignancies	I	27 participants	NCT01317927	Completed
		Breast Cancer/Prostate Cancer/Ovarian Cancer	I	25 participants	NCT04703920	Recruiting
		NSCL	I	28 participants	NCT00926640	Completed
		Multiple Myeloma	II	25 participants	NCT00131261	Completed
		Melanoma	II	32 participants	NCT05170334	Recruiting
Entinostat	Class I HDACs	Breast Cancer	I	61 participants	NCT02820961	Completed
		CNS Tumor/Solid Tumor	I II	128 participants	NCT03838042	Recruiting
		Bladder Cancer	II	20 participants	NCT03978624	Recruiting
		acute myeloid leukemia	II	24 participants	NCT00462605	Completed
		Melanoma	II	14 participants	NCT03765229	Recruiting
Givinostat (ITF2357)	Class I/II HDACs	Hodgkin’s Lymphoma	I II	24 participants	NCT00792467	Completed
Panobinostat (LBH589)	pan-DACi	Solid Tumor	I	25 participants	NCT01007968	Completed
		Lung Cancer/Head and Neck Cancer	I	44 participants	NCT00738751	Completed
Panobinostat (LBH589)	pan-DACi	Multiple Myeloma	III	767 participants	NCT01023308	Completed
		Hodgkin’s Lymphoma	III	41 participants	NCT01034163	Completed
Pracinostat (SB939)	Class I/II HDACs	Solid Tumor	I	39 participants	NCT00504296	Completed
Pracinostat (SB939)	Class I/II HDACs	Prostate Cancer	II	32 participants	NCT01075308	Completed
		AML	II	50 participants	NCT01912274	Completed
		Sarcoma	II	24 participants	NCT01112384	Completed
Romidepsin	Class I HDACs	Solid Tumors	I	18 participants	NCT01537744	Completed
		Lung Cancer	I	34 participants	NCT00037817	Completed
Romidepsin	Class I HDACs	Pancreas Cancer	I II	75 participants	NCT04257448	Recruiting
		T-Cell Lymphoma	III	271 participants	NCT01482962	Completed
Valproic Acid	Class I/II HDACs	Solid Tumors	I	36 participants	NCT00529022	Completed
		NSCL	I	25 participants	NCT00084981	Completed
		AML	I II	36 participants	NCT00995332	Completed
		Cervical Cancer	II	18 participants	NCT00404326	Completed
Valproic Acid	Class I/II HDACs	Glioma	III	167 participants	NCT03243461	Recruiting
		Childhood Ependymoma	III	480 participants	NCT02265770	Recruiting
Vorinostat	Class I/II HDACs	Solid Tumors	I	28 participants	NCT00121277	Completed
		Gastric Cancer	I II	45 participants	NCT01045538	Completed
		Mesothelioma	III	661 participants	NCT00128102	Completed
		Multiple Myeloma	III	637 participants	NCT00773747	Completed
		AML	III	754 participants	NCT01802333	Completed
CHR-3996	HDAC	Solid Tumors	I	40 participants	NCT00697879	Completed
Chidamide	Class I HDACs	Cervical Cancer	I	40 participants	NCT04651127	Recruiting
		AML	I II	250 participants	NCT03031262	Recruiting
Chidamide	Class I HDACs	Sarcoma	II	53 participants	NCT04025931	Recruiting
		Neuroendocrine Tumors	II	23 participants	NCT05113355	Recruiting
Quisinostat	HDACs	NSCL	I	51 participants	NCT02728492	Completed
		Lymphoma	I	92 participants	NCT00677105	Completed
		Multiple Myeloma	I	18 participants	NCT01464112	Completed
AR-42	Pan-DAC Inhibitor	Plasma Cell Myeloma	I	9 participants	NCT02569320	Completed
		AML	I	13 participants	NCT01798901	Completed

This table includes agents targeting for surface markers, transcriptional factors, glucose, glutamine, lipid, the Shh, NOTCH, WNT/β-catenin, TGF-β and JAK/STAT signaling pathways, DNA modification (DNMT inhibitors) and histone modification (HDAC inhibitors) of DNA

*N/A* Not Applicable

### Agents targeting cell surface markers of CSCs

As specific biomarkers have been discovered at the surface of multiple cancer cells, they have already become common therapeutic targets in cancers, especially to identify and eradicate CSCs. Among them, CD123, CD44v6 and EpCAM are three common biomarkers of CSCs that are frequently utilized as therapeutic targets. Notably, the choice of targets can vary depending on the specific expression patterns of CSC surface markers in different types of tumors (NCT03869190, NCT02674763, NCT04430530 and NCT04216524).

IMGN632 has received a breakthrough agent designation from the FDA for the treatment of plasmacytoid dendritic cell tumors, targeting CD123 [347]. Subsequently, a study in acute lymphoblastic leukemia (ALL) showed a positive therapeutic effect of IMGN632 on B-ALL, characterized by high CD123 levels [348]. Many other agents could also recognize CD123 and then eradicate CSCs, such as talacotuzumab, flotetuzumab and tagraxofusp [349–351]. Furthermore, bivatuzumab mertansine is a humanized anti-CD44v6 monoclonal antibody (mAb) used for HNSCC treatment, but it has been associated with severe agent-related adverse effects [352, 353]. Catumaxomab is a specific EpCAM antibody used in the treatment of various solid tumors, with a focus on targeting CSCs [354–357].

### Agents targeting transcriptional factors of CSCs

Oct4, Sox2, c-Myc, KLF4, Nanog, and PBX1 are specific transcription factors (TFs) associated with CSCs, and they have been considered as potential therapeutic targets for CSCs. While it is challenging to develop agents that target TFs, effective efforts have been made in preclinical models. For instance, Ivermectin, a polycyclic lactone pesticide, has been reported as an inhibitor with inhibitory effects on CSCs by targeting Oct4, Sox2, and Nanog [335]. Increasing evidence indicates that several inhibitors such as ZF-5985KD and Peptide aptamer P42 inhibit CSCs by targeting Sox2 [336]. Yangling Li et al. and Ruosi Yao et al. demonstrated respectively that statins significantly downregulated KLF4 and D347-2761 blocked c-Myc in CSCs derived from osteosarcoma [338, 339]. Moreover, T417 represents a novel potential agent that targets PBX1, thereby inhibiting CSCs in ovarian cancer [340].

### Agents targeting the metabolism of CSCs

Metabolism is intricately linked to the TME and plays a crucial role in controlling CSCs. Emerging research has identified metabolic agents as potential therapeutic agents for targeting CSCs in a specific manner. These novel therapeutic agents in numerous preclinical and clinical studies can be categorized into two main groups: those that aim to hinder the metabolic characteristics of CSCs, such as glucose inhibitors, glutamine inhibitors, and lipid inhibitors, and those designed to alleviate hypoxia, such as HIF inhibitors.

Glucose inhibitors targeting glycolysis have demonstrated effectiveness. For instance, the antidiabetic medication Metformin has been reported to attenuate glycolysis in HCC [341]. Additionally, Michael et al. demonstrated that 2-deoxy-D-glucose reduced the proliferation of CSCs in colon cancer [358]. Glutamine represents another potential metabolic target for CSCs, and several glutamine inhibitors have been developed. For example, R-HepG2 has shown effectiveness in targeting glutamine in the treatment of CSCs in HCC [234]. Furthermore, CB-839 is a glutaminase inhibitor that has demonstrated clinical therapeutic efficacy in lung cancer [359]. Lipid inhibitors, such as omeprazole and cerulenin, have also been discovered with the potential to treat CSCs [360, 361]. On the other hand, increasing evidence suggests that HIF inhibitors, such as PT2385 and 32-134D, have therapeutic effects in various cancers by targeting CSCs [346, 362].

### Agents targeting CSC-associated signaling pathways

Targeting the signaling pathways involved in the regulation of CSC characteristics has become a comprehensive key technology for cancer therapy. Currently, the main related signaling pathways include the Notch, WNT/β-catenin, Shh, TGF-β and JAK/STAT3 signaling pathways. These pathways not only act independently but also interact with one another to maintain CSC characteristics.

#### Notch signaling pathway inhibitors

As mentioned before, the Notch signaling pathway is of the utmost importance in maintaining CSC characteristics. The tumor-promoting function of Notch signaling has been shown in glioma, colon cancer, breast cancer, gastric cancer, and myeloma [363]. Moreover, breakthroughs have been made in Notch-targeted cancer therapies through three major classes of Notch pathway inhibitors, including γ-secretase inhibitors, Notch receptor antibodies, and Notch ligand antibodies.

For instance, RO4929097 is a γ-secretase inhibitor with a high affinity for Notch signaling [364]. At present, over 30 clinical trials have used RO4929097 as an antitumor agent in various solid tumors (NCT01131234), sarcoma (NCT01154452) and melanoma (NCT01196416). In addition, MK-0752 shows well-tolerated antitumor activity against CSCs in breast cancer by inhibiting γ-secretase (NCT00645333) [365]. Additional selective γ-secretase inhibitors, including LY3039478 (NCT02836600), AL101 (NCT03691207), and BMS-906024 (NCT01292655), are currently undergoing clinical trials. Blocking Notch signaling through the inhibition of Notch receptors and ligands represents another strategy. Brontictuzumab is a Notch1 mAb, and its notable clinical benefits in Notch1-mutated adenoid cystic carcinoma (ACC) patients have been documented [366]. ABL001, on the other hand, targets DLL4, a prominent Notch ligand, thereby impeding angiogenesis in gastric cancer and colon cancer, while also reducing the population of CSCs [367, 368].

#### WNT/β-catenin signaling pathway inhibitors

Activation of WNT/β-catenin signaling in CSCs contributes to maintaining the characteristics of CSCs, promoting tumor processes and poor patient prognosis. At present, agents targeting the β-catenin and frizzled molecules of the WNT/β-catenin signaling pathway have been under clinical trials.

CWP232291 is a novel small molecule β-catenin inhibitor that aims to suppress β-catenin and potentially achieve clinical remission in prostate cancer [369]. Vantictumab and ipafricept have shown effectiveness in breast, ovarian, and pancreatic cancers by targeting and blocking frizzled receptors [370]. Additionally, other WNT/β-catenin signaling inhibitors have participated in various ongoing clinical trials, including PRI-724 (NCT01606579), OMP-54F28 (NCT01608867 and NCT02092363), ETC-1922159 (NCT02521844) and LGK974 (NCT01351103).

#### Shh signaling pathway inhibitors

Aberrant Shh signaling has been proven in various types of cancer. SMO is the most important component of the Shh signaling pathway that mediates TF transfer. Consequently, targeting SMO has become a primary strategy for inhibiting Shh signaling. It’s worth noting that SMO inhibitors exhibit greater effectiveness in treating basal cell carcinoma (BCC) and medulloblastoma compared to other cancer types [371].

Vismodegib is an FDA-approved SMO inhibitor for the treatment of advanced BCC. An American clinical trial indicated that 73% of BCC patients enrolled in the clinical trial had tumor shrinkage after vismodegib treatment (NCT00833417) [372]. However, the therapeutic effect of vismodegib was not satisfactory in other solid tumors (NCT01064622, NCT01209143). Increasing evidence has demonstrated that glasdegib contributes to a good therapeutic effect on AML by inhibiting Shh signaling (NCT01546038) [373, 374]. Other Shh inhibitors, such as BMS-833923, LDE225 and LEQ506, are also under investigation in clinical trials for various cancer treatments, and the effect remains to be confirmed.

#### Other signaling pathway inhibitors

Activation of the TGF-β signaling pathway and JAK/STAT3 signaling pathway has also been found in CSCs. TGF-β is a tumor promoter; therefore, blocking TGF-β has been a novel strategy in cancer therapy [375]. Vactosertib, a well-tolerated small molecule TGF-β inhibitor, has been tested in clinical trials of multiple cancer types (NCT03143985, NCT02160106). In addition, fresolimumab, galunisertib and AVID200 are other selective TGF-β inhibitors designed for various cancers [376]. In the JAK/STAT3 signaling pathway, AZD4205 and ruxolitinib have been discovered to be effective in the treatment of solid tumors and lymphoma [377–379]. Other agents related to CSC-associated signaling pathways in clinical trials are listed in Table 1.

### Agents targeting epigenetic modifications

Epigenetic modification has garnered significant interest as a crucial component of the regulatory networks governing CSCs. Currently, epigenetic agents play vital roles in combatting CSC characteristics and targeting the overall tumor population. Two extensively researched epigenetic agents, DNMT inhibitors and HDAC inhibitors, are subjects of ongoing clinical cancer trials. In this section, we review agents that target epigenetic modifications, with a particular focus on DNMT and HDAC inhibitors, across various cancer types.

DNMTs are essential enzymes involved in DNA methylation, which in turn modulates CSC stemness. Decitabine and azacitidine are two major DNMT inhibitors used for cancer treatment. Decitabine is an FDA-approved DMNT inhibitor that has been applied in myelodysplastic syndrome, AML and solid tumors [380–382]. Azacitidine is another extensively studied DNMT inhibitor in clinical settings. Increasing evidence indicates its good tolerability and efficacy in AML, particularly among older patients [4, 383]. Furthermore, SGI-110, disulfiram, and Aza-TdC are additional DNMT inhibitors currently undergoing clinical trials for various cancer types.

HDACs remove acetyl groups, resulting in tighter binding between DNA and histones. Consequently, HDAC inhibitors have the potential to induce cell apoptosis [384]. To date, it has been found that givinostat can function in the treatment of Hodgkin’s lymphoma [385]. Vorinostat and romidepsin are two HDAC inhibitors specifically used for the treatment of cutaneous T-cell lymphoma (NCT01728805, NCT0148296). Moreover, a phase III clinical trial launched by Dr. Kim showed that a novel agent, mogamulizumab, significantly prolonged the progression-free survival of cutaneous T-cell lymphoma patients [386]. Apart from these agents, other HDAC inhibitors, such as belinostat, panobinostat, and chidamide, are also important in antitumor and anti-CSC therapies [387].

Moreover, more agents targeting CSC-associated regulatory networks and their clinical status can be found in Table 1.

## Conclusions and perspectives

CSCs represent a subpopulation of stem cells characterized by their self-renewal capabilities and differentiation potential, contributing significantly to cell proliferation, metastasis, and tumor growth. The regulatory networks governing CSCs encompass transcriptional control, post-transcriptional control, epigenetic modifications, control by the tumor microenvironment (TME), and regulation by the epithelial-mesenchymal transition (EMT) process. This review also explores the roles of Notch, WNT/β-catenin, Sonic hedgehog (Shh), TGF-β, and JAK-STAT3 signaling pathways in CSC regulation. Additionally, several factors employed by CSCs are closely associated with therapy resistance. Promisingly, a variety of CSC-targeted therapies have been developed and are currently undergoing clinical trials, offering a hopeful outlook for the future of cancer treatment.

However, effectively eradicating CSCs faces several challenges. Firstly, the complete identification of surface markers specific to CSCs remains elusive, as CSCs can adapt by altering their surface markers to evade immune responses. Secondly, most current studies have isolated CSCs from the tumor microenvironment, which limits our understanding of how the tumor microenvironment influences CSCs, a crucial aspect of actual tumor development. Thirdly, irrespective of transcription factors, signaling pathways, or RNA and epigenetic modifications, there are regulatory networks that control both CSCs and normal cell physiological activities, posing limitations on targeted cancer therapies. Lastly, there is currently no effective therapy available for targeting the quiescent state of CSCs.

## Data Availability

All clinical trials mentioned in this current review are available in *ClinicalTrials.gov* (https://clinicaltrials.gov/); The figures in this article were created with BioRender.com.

## Abbreviations

ACC: Adenoid cystic carcinoma
ADAR: Adenosine deaminase
AGO2: Argonaute2
ALDH: Aldehyde dehydrogenase
AML: Acute myeloid leukemia
ASC: Adult stem cell
A-to-I: Adenosine-to-inosine
AZIN1: Antizyme inhibitor 1
BCC: Basal cell carcinoma
CAF: Cancer-associated fibroblasts
CDK13: Cyclin-dependent serine/threonine protein kinase 13
CML: Chronic myeloid leukemia
CSC: Cancer stem cell
EMT: Epithelial-Mesenchymal Transformation
EMT-TF: EMT-inducing transcription factor
5-FU: 5-Fluorouracil
GRIA2: Glutamate receptor subunit B
HA: Hyaluronic acid
HCC: Hepatocellular carcinoma
HGF: Hepatocyte growth factor
HIF: Hypoxia-inducible factor
HNSCC: Head and neck squamous cell carcinoma
ISG15: Interferon-stimulated gene 15
LGR5: Leucine-rich repeat containing G protein-coupled receptor
LncRNA: Long noncoding RNA
m^6^A: N6-methyladenosine
mAb: Monoclonal antibody
m^5^C: 5-Methylcytosine
MDR: Multidrug resistance
MiRNA: MicroRNA
METTLE: M^6^A methyltransferase
NICD: Notch ICD
MSC: Mesenchymal stem cell
NSCLC: Non-small cell lung cancer
PODXL: Podocalyxin-like
QKI-5: Quaking gene 5
RBP: RNA-binding protein
ROS: Reactive oxygen species
Shh: Sonic hedgehog
SOCS2: Suppressor of cytokine signaling 2
SOD2: Superoxide dismutase
Sox2: Sex determining region Y-Box-2
T-ALL: T-cell acute lymphoblastic leukemia
TAM: Tumor-associated macrophage
TME: Tumor Microenvironment
TNBC: Triple-negative breast cancer
TF: Transcription Factor
YAP1: Yes-associated protein 1
